# An Overview of the Supramolecular Systems for Gene and Drug Delivery in Tissue Regeneration

**DOI:** 10.3390/pharmaceutics14081733

**Published:** 2022-08-18

**Authors:** Saketh Reddy Ranamalla, Alina Silvia Porfire, Ioan Tomuță, Manuela Banciu

**Affiliations:** 1 Department of Pharmaceutical Technology and Bio Pharmacy, Faculty of Pharmacy, “Iuliu Hațieganu” University of Medicine and Pharmacy, 400010 Cluj-Napoca, Romania; 2 Doctoral School in Integrative Biology, Faculty of Biology and Geology, “Babeș-Bolyai” University, 400015 Cluj-Napoca, Romania; 3 Department of Molecular Biology and Biotechnology, Center of Systems Biology, Biodiversity and Bioresources, Faculty of Biology and Geology, “Babeș-Bolyai” University, 400015 Cluj-Napoca, Romania

**Keywords:** supramolecular, self-assembling, non-covalent interactions, hydrogels, nanofibers, drug delivery, gene delivery, tissue regeneration

## Abstract

Tissue regeneration is a prominent area of research, developing biomaterials aimed to be tunable, mechanistic scaffolds that mimic the physiological environment of the tissue. These biomaterials are projected to effectively possess similar chemical and biological properties, while at the same time are required to be safely and quickly degradable in the body once the desired restoration is achieved. Supramolecular systems composed of reversible, non-covalently connected, self-assembly units that respond to biological stimuli and signal cells have efficiently been developed as preferred biomaterials. Their biocompatibility and the ability to engineer the functionality have led to promising results in regenerative therapy. This review was intended to illuminate those who wish to envisage the niche translational research in regenerative therapy by summarizing the various explored types, chemistry, mechanisms, stimuli receptivity, and other advancements of supramolecular systems.

## 1. Introduction

Tissue regeneration has been an area of prominent research in the twenty-first century that emphasizes the regeneration of human cells, tissues, or whole organs for reestablishing structural and physiological functions [1]. The targeted physiological areas of this interdisciplinary research field are widespread, including dermal, cardiovascular, musculoskeletal, gastrointestinal, and nervous systems, and most of the medications being developed are cell and gene therapy-based medications [2]. In most scenarios such as injuries where regenerative strategies are followed, the extracellular matrix is either partly or completely lost [3]. The extracellular matrix (ECM), which is a three-dimensionally arranged interwoven network of fibers derived from the proteins, proteoglycans and glycosaminoglycans act as structural supports and as media for the transfer of oxygen and nutrients [4]. This ECM is highly dynamic and undergoes remodeling and serves the purpose of being a tissue-specific substrate that allows cell attachment, migration, and proliferation. In the cases of injuries or pathological conditions, the ECM acts as a base for the signaling molecules such as growth factors and morphogens, thereby creating a microenvironment for the cells to differentiate and function [5].

In conditions where ECM is lost, the design and manufacturing of a similar mechanistic scaffold that can simulate the biological environment of the damaged tissues is a major challenge. The biomimetic scaffolds should be able to either reproduce the ECM or be able to substitute it by providing the required physical support that is usually provided by the various proteins for the cells and the signaling molecules to regenerate or repair the part of the tissue. The scaffolds should also be capable of supporting the normal functioning of the targeted tissue and improve the quality of life for the patients.

Biomolecules such as proteins, receptors, enzymes, and many small molecules have often been observed to self-assemble themselves, and to form supramolecular complexes that allow them to regulate a variety of biological functions [6]. This phenomenon of self-assembly backed up by non-covalent intermolecular forces is attributed to Supramolecular chemistry coined by Jean-Marie Lehn, who received the Nobel prize in 1987 with Donald J. Cram and Charles J. Pedersenn for their contribution to the field. He defined it as the “chemistry beyond the molecule”, where molecules are held together by non-covalent interactions such as hydrogen bonds, van der Waals forces, and ion or dipole interactions [7].

Attributed to this non-covalent chemistry, many supramolecular bio-functional materials have been developed in the past three decades for regenerative medicine. These materials, while acting as a mechanistic scaffold, are aimed to deliver therapeutic molecules/genes and at the same time try to mimic the functional properties of the tissue. These bio-functional materials have proven advantages of safety, response to external stimuli, reversibility, biomimicry, and many other benefits in in vitro and in vivo conditions [8]. Researchers have reviewed both the scaffold and scaffold-free approaches that mimic the biological processes for regenerative medicine taking in account of the spatiotemporal data that occur in in vitro organogenesis and in vivo conditions [9,10]. With this due diligence, the researchers have instituted confidence in this platform and are working towards making technological advances and bringing these systems closer to the patients [11]. This article uniquely reviewed the supramolecular systems that have been applied for tissue regeneration with their types based on origin, fundamentals of mechanism, technical aspects of synthesis and characterization, and innovations to improve clinical applicability.

## 2. Types of Supramolecular Systems Used for Tissue Regeneration

**Figure 1:**
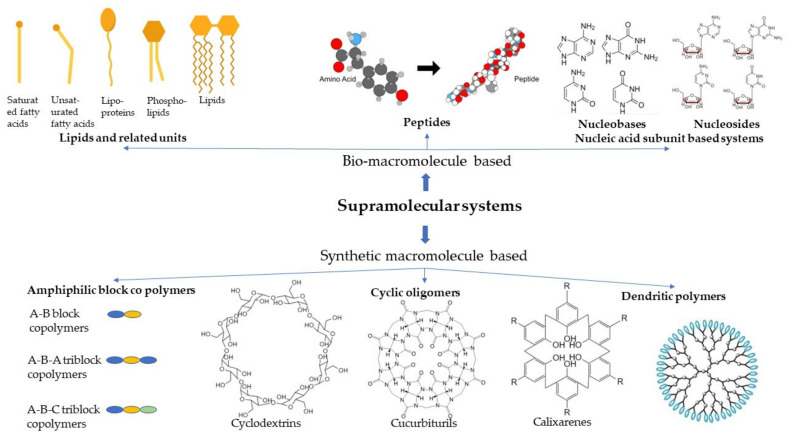
Types of supramolecular systems used for tissue regeneration (created with Biorender.com [accessed on 30 June 2022]).

There has been a plethora of work carried out on supramolecular systems owing to their tunability and adaptability for regenerative medicine. For the sake of simplicity, we classified the systems into two groups based on their origin: bio-macromolecule-based and synthetic-based systems, as shown in Figure 1. In addition to these systems, there are also supramolecular systems derived from the natural biomaterials.

### 2.1. Bio-Macromolecule-Based Systems

**Figure 2:**
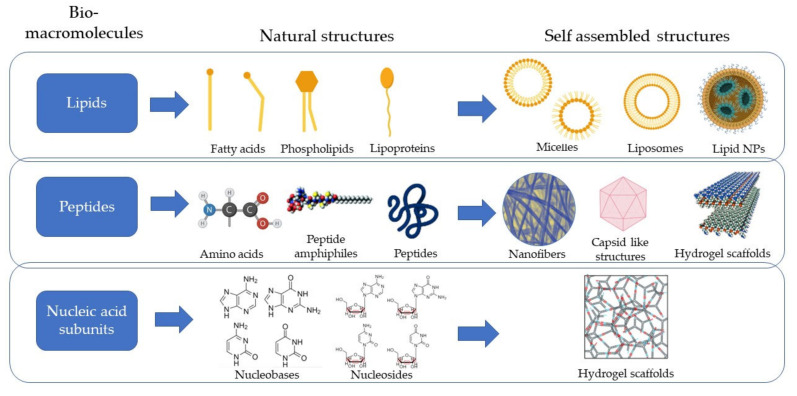
Biomacromolecule based self-assembly structures (created with Biorender.com [accessed on 30 June 2022]).

This category of supramolecular systems uses the advantage of natural biomimicry offered by the materials as they are also inherently synthesized and circulated amongst the cells and tissues. Based on the type of macromolecules used, it can be further classified into lipids, peptides, and nucleic acid-based systems, as illustrated in Figure 2.

#### 2.1.1. Lipid-Based Systems

Glycerides and fatty acids are the structural units of the lipids and usually do not form self-assembling structures until the former ones are in an aqueous environment, but the lipids, due to their significant hydrophobicity, tend to form supramolecular systems much more easily. The lipids can be modified to form supramolecular delivery systems such as nanoparticles [12], micelles [13], liposomes [14], hydrogels [15], and dendrimers [16]. Lipids, in combination with other bio-macromolecules such as nucleic acids [17] and proteins [12], tend to act as a scaffold in the place of the lost tissue in the regenerative medicine domain. These supramolecular-based systems are used majorly for the delivery of drugs and genetic material to the targeted tissues.

Henrich et al. reported the synthesis of soft-core nanoparticles with a diameter of about 10 nm that are similar in size, shape, composition, protein structure and surface chemistry to human High-Density Lipoproteins (HDL). These self-assembled phospholipid particles can carry out most of the HDL functions such as those of the cholesterol efflux from macrophages, cholesterol delivery to hepatocytes, lecithin: cholesterol acyltransferase activity support, and inflammation suppression. These synthetic HDLs have immense potential to function as therapeutic agents [12]. Additionally, utilization of biologically active monoglycerides such as glycerol monolaurate (GML) or its ether derivative 1-O-dodecyl- rac -glycerol (DDG) to form supramolecular assemblies producing nano micelles was reported by Yoon et al. These micelles can modulate the membrane interactions with cell-membrane-mimicking giant unilamellar vesicles that can assist in nanomedicine [13].

Liposomes are self-assembled phospholipid membranes that can deliver therapeutically active compounds which could be drug molecules, cells, growth factors, genetic material, and others. Tremendous research involving liposomes has been documented in the last three decades and a lot of modifications have been made to the structure of the liposomes to regulate the release of the therapeutic materials. Hu et al. in their research work on the poly(lactic-co-glycolic acid)-liposomes delivered both microRNA 145 and the platelet-derived growth factor, which helped in the cell differentiation of the mesenchymal stromal cells into vascular smooth muscle cells. Though the mesenchymal stromal cells have differentiation ability, the efficiency of differentiation is low. However, the research team could translate these results in the in vivo skin wound healing models using male Sprague–Dawley rats, providing new insights into utilizing the liposomes as a multi-delivery system [18].

Liposomes also have been integrated onto biomaterial scaffolds to release drug or genetic material load to be dually efficient as a mechanical support and to achieve the desired therapeutic effect. Lee et al. demonstrated the use of liposomes loaded with smoothened agonists for the Hedgehog signaling pathway responsible for endochondral bone healing. These liposomes are then adhered to using biocompatible polydopamine adhesive onto the trabecular bone mimetic apatite-coated scaffolds. The in vitro experiments in the mouse bone marrow stem cells and the in vivo experiments using the parietal bone defect model have suggested successful cell adhesion and cell proliferation [19]. Another similar example is from the research by Mohammadi et al., where the team achieved a sustained release of the BMP-2 peptide over 21 days from the liposomes attached to the poly L-lactic acid nanofibers coated with hydroxyapatite nanoparticles. The in vitro studies have confirmed the cell proliferation and cell differentiation of the mesenchymal stem cells. The in vivo osteoinduction efficiency was evaluated by the subcutaneous implantation of the scaffolds in the in vitro rat models, which revealed primary ossification with the liposomes [20].

#### 2.1.2. Peptide-Based Systems

Peptides are unique biomolecules placed between the small molecules (<500 Da) which could be easily developed, and the large molecules (>5000 Da) can be specific and potent at the same time [21]. Tissue regeneration, in a sense, necessitates the use of two complementary vital elements. The first element is a physiologically compatible scaffold that could be easily absorbed by the body without causing injury, and the second is appropriate cells, such as stem cells and primary cells, which can successfully replace damaged tissues without causing harm. It would be beneficial for tissue healing if suitable biological scaffolding could be used to drive cell differentiation. Many scaffold-forming self-assembling peptides have been examined for this purpose, with peptide amphiphiles being one of the most well-studied groups [22].

Self-assembling peptide amphiphiles consist of these hydrophilic and hydrophobic moieties that tend to form secondary structural motifs such as β-sheets and α-helices [23]. These secondary structures are backed by hydrogen bonding and van der Waals forces and the electrostatic associations assemble to form 1 D nanostructures. When the amphiphilic structures are placed in water, the hydrophobicity of aliphatic chains triggers the formation of lower surface area and high-aspect-ratio 3 D structures such as nanofibers. Peptide nanofibers have been researched and been employed for the regeneration of every tissue in the body, owing to their promising results in mimicking the complex organization of tissues [24]. One such application was studied by Mansukhani et al., who investigated the use of peptide amphiphile nanofibers containing the apolipoprotein A1 (Apo A1)-derived targeting peptide 4F as nanocarriers for Liver X receptor (LXR) delivery (ApoA1-LXR PA) in vivo and showed that supramolecular nanostructures can be used as safe and effective drug nanocarriers in the treatment of atherosclerosis [25].

The supramolecular behavior of the peptides has also been advantageous in designing the nanocarrier systems in the form of dendritic structures which can engulf the drug or genetic material and deliver it to selective tissue. Li et al. used the self-assembly of poly(L-lysine) dendrimers to form pH-responsive capsid-like nanocarriers which could enhance the penetration and accumulation of doxorubicin in multicellular tumor spheroids mimicking solid tumor tissues [26].

Any biomaterial that is being developed for tissue regeneration should be physically and chemically equivalent to that of the extracellular matrix of the tissue [27]. Peptides, peptide conjugates, and peptide aggregates, due to their supramolecular capability, better biocompatibility, and biodegradability than the synthetic supramolecular systems, are the preferred scaffolds by researchers [28]. They are preferred over polymeric scaffolds due to the ability of angiogenic sprouting and vascularization. This was extensively studied by Siddiqui et al. in their research article, where microporous polymeric scaffolds and viscoelastic nano porous peptide hydrogels were used to create two-component scaffolds. The implant vascularization and cellular infiltration were measured by the bioactive moieties in the primary sequences of the peptide monomers, hence showing the utility of soft supramolecular peptide hydrogels for designing multi-component regenerative scaffolds [29].

Amino acids such as tyrosine and phenylalanine form hydrogels readily through π–π interactions and are being majorly used for external stimuli-triggered delivery systems. Just like the naturally occurring amino acids, there are synthetic amino acids that form small molecular hydrogels such as diphenylalanine which undergo the π–π stacking of amino acids to form the hydrogels. The utilization of self-assembling peptides to form such hydrogels through non-covalent interactions helps in delivering biologics such as antibodies, growth factors, genes etc. [30]. Abraham et al. showed, in their research article, the improved versions of the Phenylalanine-derived gelators that are protected by Fluorenyl methoxycarbonyl (Fmoc) form some of the most stable hydrogels from the low molecular weight gelators [31]. Synthetic amino acids such as diphenylalanine also have been used in combination with some cyclic macromolecules such as β-cyclodextrin to form supramolecular nanospheres that can be reversibly assembled and disassembled into nanofibers. Zhang et al. used ferrocene-modified diphenylalanine that can reversibly assemble and dissemble by the chemical redox reactions of ferrocene groups, therefore demonstrating a redox-sensitive peptide supramolecular system [32].

#### 2.1.3. Nucleic Acid Subunit-Based Systems

The building blocks of the nucleic acids such as nucleosides and nucleotides have recently been widely explored as diagnostics, tissue scaffolding, and targeted drug release systems due to their capability of self-assembly. All the nucleosides and nucleotides contain the nucleobases and ribose or a deoxy ribose sugar molecule. Nucleosides are differentiated from the nucleotides by consisting of phosphate groups in addition to the nucleobases and the ribose sugar molecule. The nucleobases are nitrogen-containing heterocyclic compounds which can be purines (Adenine {A} and Guanine {G}) and pyrimidines (Uracil {U}, Thymine {T}, and Cytosine {C}). Due to their aromatic nature, these nucleobases naturally exhibit π–π stacking and, additionally, there are hydrogen bond acceptors and donors in both types of nucleobases favoring hydrogen bonding. These non-covalent interactions, called Watson–Crick base pairing and Hoogsteen base pairing, help to form the supramolecular systems. These nucleobases are attached to a ribose sugar molecule via glycosidic linkages which are further connected to a phosphoryl group constituting the nucleic acids DNA and RNA. The phosphoryl group bears an anionic charge which further strengthens the supramolecular system by electrostatic interactions [33].

The nucleic acid subunit-based systems tend to form gels when supramolecular assembly is induced. The induction process typically takes place in the presence of lipophilic groups which increase the hydrophobicity appending the self-association. Among these types, guanosine and its derivative-based organogels have been much focused on lately. Zhao et al. have carried out immense research on the supramolecular nucleoside-based hydrogels and reported on the applications in drug delivery, regenerative medicine, and theranostic devices. Zhao et al. used Isoguanosine and its derivatives such as the 2′-deoxyribonucleoside and the 2′-deoxy-2′-fluoro ribonucleoside to form hydrogels in alkali metal salt solutions, which had excellent long-term stability with good loading efficiencies for small molecules [34]. Their recent article mentioned the dual functional supramolecular hydrogel made from Isoguanosine-borate-guanosine prepared by a one-pot procedure that can be used for small molecule delivery [35]. Other derivatives of adenine and cytosine have also been employed, but little literature is available on the application for regenerative medicine [36]. Cheng et al. invented self-assembling physically cross-linked supramolecular polymers composed of cytosine, poly(ethylene glycol) (PEG), and hydrophobic poly(epichlorohydrin). They observed that the cytosine functionalized substrates improved wound healing by promoting quick cell migration into the injured cellular surface [37].

### 2.2. Synthetic Supramolecular Systems

**Figure 3:**
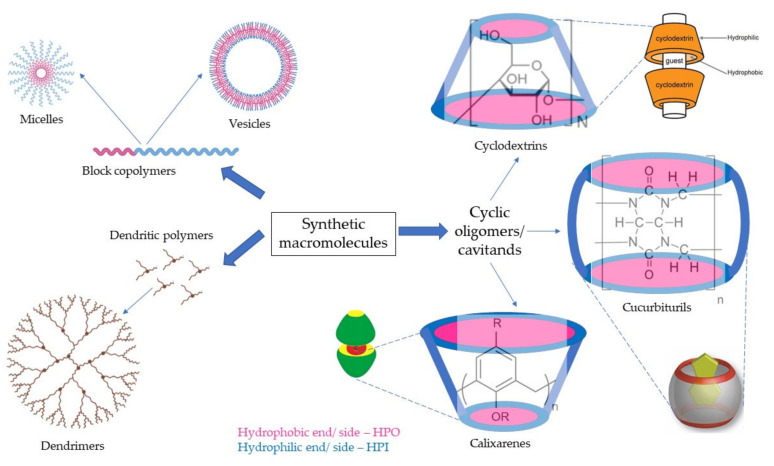
Sub-types of synthetic macromolecule-based supramolecular systems and their self-assembly structures (created with Biorender.com [accessed on 30 June 2022]).

These groups of synthetically produced macromolecules that are being used for tissue regeneration mostly include block copolymers, dendritic polymers, or cyclic oligomers. The subtypes are diagrammatically represented in Figure 3. Block copolymers that are usually used for regenerative medicine comprise diblock, triblock, and other multiblock copolymers, whereas cyclic oligomers comprise cyclodextrins, calixarenes, and cucurbit(8)urils. Then there are dendritic polymers, which are mostly classified by the type of subunits that comprise them. The synthetic supramolecular systems have flexibility over biomacromolecule-based systems for being able to be manufactured as required by the tissue to be mimicked.

#### 2.2.1. Block Copolymers

Block copolymers, as the name suggests, consist of a linear arrangement of blocks of two or more polymers connected end-to-end. Based on their sequential arrangement of blocks, they can vary from the di-block (A-B) consisting of two blocks, to tri-block (A-B-C or A-B-A) consisting of three blocks, to multi-block (A-B)_n_ consisting of multiple blocks. The synthesis of these block copolymers is performed by polymerization techniques utilizing ions, free radicals, and metal catalysts. The polymers are then electro spun into nanofibers or are employed in techniques such as bio-printing to form tissue scaffolds. The block copolymers majorly act as substitutes for the natural scaffold, possessing most of the necessary characteristics such as porosity for cell attachment, angiogenesis, nutrients transfer, biodegradability, and biocompatibility [38].

Rico et al. listed the utility of poly(ethylene oxide)-poly(propylene oxide)-poly(ethylene oxide) tri-block copolymers for gene delivery in human regenerative medicine. They reviewed the formation of micellae and hydrogels, their applications, and their limitations [39]. Other ABA tri-block copolymers such as poly(propylene fumarate)-polyethylene glycol-poly(propylene fumarate) have been used for three-dimensional (3D) printing of amphiphilic hydrogels with demonstrated mechanical stability and tolerance for degradation [40].

These amphiphilic block copolymers can also be made stimuli-responsive to physiological conditions, making them more functional for tissue regeneration. Lee et al. synthesized poly(ε-caprolactone) block copolymers and proposed them as a model for drug and gene delivery with demonstrated thermo-responsive phase transition and in vitro biodegradability [41]. Biodegradable amphiphilic poly (ethylene glycol) (PEG)-based ether-anhydride terpolymer was synthesized to form different-shaped self-assembling micelles and they were assessed for cellular internalization rate. Through this research, Yang et al. found that comb-shaped micelles had a high blood circulation rate, thereby acting as a suitable drug carrier system for regenerative medicine [42].

#### 2.2.2. Dendritic Polymers

Dendrimers are a class of synthetic polymers that have evolved over the past two decades. The term is derived from Greek, which means Dendra—tree and meros—part of which suggests the architecture of these polymeric macromolecules that resemble a tree. As in a tree, several branching units originate from a multi-functional core unit and end with a capping unit. These repetitive branching monomer units of the synthetic macromolecules from the core are organized into layers called generations. These systems can be surface modified for many applications including reducing the cytotoxicity, clearance, and increasing biodistribution. Among the wide variety of dendrimers being researched, glycodendrimers and peptide dendrimers are found to be more predominant. The glycodendrimers are derived from the repetitive carbohydrate units and the peptide dendrimers have a peptidyl core with functional branching units. In contrast with the polymers acting as a natural scaffold, they act as efficient delivery systems for small molecules, proteins, and genetic material [43]. To form the scaffold, they use non-covalent interactions such as hydrogen bonds, van der Waals forces, and electrostatic interactions in addition to covalent bonds [44].

Bai et al. reported a self-reinforcing hydrogel for bone repair by utilizing non-covalent interactions between β-cyclodextrin-graft-poly(N-isopropyl acrylamide) as host and adamantane decorated generation 2.5 poly(amidoamine)s dendrimer as guest polymer. Furfuryl amine grafted chondroitin sulfate (ChS-F) and maleimido-terminated poly(ethylene glycol) (PEG2K–AMI) were then chemically cross-linked to the hydrogel to obtain a scaffold with high water content and mechanical strength [45]. Dendrimer nanoparticles made up of carboxymethyl chitosan/poly(amidoamine) were used to observe the cell internalization in neurons, astrocytes, oligodendrocytes, and microglial cells using fluorescent probes. These systems can be further used for the delivery of drugs to the damaged parts of the central nervous system [46].

The delivery of genetic material by dendritic systems has also been a widely researched topic. Bi et al. proposed hydrogels made up of poly(amidoamine) (PAMAM), hyaluronic acid (HA) and arginyl glycyl aspartic acid (RGD) peptide with reliable results in cell viability, proliferation, and attachment demonstrated in vitro in the bone marrow stem cells [47]. Zhu et al. used dendrimers for the delivery of a single plasmid construct carrying Yamanaka factors that can generate pluripotent stem cells from mouse embryonic fibroblasts [48]. Other plasmid DNA was shown to be delivered efficiently with functionalized generation 5 (G5) dendrimers of PAMAM with hydrophobic chains that have reduced cell cytotoxicity in mesenchymal stem cells (MSC) [49].

Researchers have also come up with new strategies such as the layer-by-layer (LbL) technique for the supramolecular dendrimers that can be used for the inclusion and modulation of the release of pharmaceutical compounds or various bioactive substances [50].

#### 2.2.3. Cyclic Oligomers

Cyclic oligomers are the class of synthetic self-assembly systems that consist of cyclic polymers that use non-covalent interactions to form container-shaped structures. These structures are also called cavitands and allow them to act as hosts for many guest molecules including small and macromolecules. The most widely used supramolecular cyclic oligomers for regenerative medicine are cyclodextrins, calixarenes, and cucurbiturils [51].

Cyclodextrins (CD) are a group of oligosaccharides arranged cyclically to have a hydrophobic cavity and a hydrophilic external face. They are formed by the enzymatic conversion of starch and are named α, β, and γ cyclodextrins constituting 6,7 and 8 glucose subunits linked by α-1,4-linkages respectively [52]. Cyclodextrin use in regenerative medicine has been an extensively researched topic with a wide range of applications ranging from serving as scaffolds for cartilage, bone, and other tissues to vehicle/vectors for genes and drugs. For regeneration of cartilaginous tissues and wound healing, a combination of CD (especially β-CD) with hyaluronic acid (HA) has been widely investigated. For example, Zhao et al. studied the photo-responsive reversible supramolecular interactions between the azobenzene and these complexes cyclodextrin-bearing hyaluronic acid host polymer (HA-CD) for wound healing [53]. Zhang et al. prepared the biocompatible supramolecular polymeric nanofibers that comprise a network of the HA-CD and magnetic field facilitated self-assembly of magnetic nanoparticles made up of adamantane and actin-binding peptide. These nanofibers are capable of cellular internalization and polarizing mammalian cells in the presence of external magnetic field [54]. CD can also play the role of stabilizers in the supramolecular systems as demonstrated by Milcovich et al., who used β-CD as a stabilizer for unilamellar catanionic vesicles, which could deliver bioactive molecules [55].

Some researchers have used the host–guest chemistry of these macromolecules co-polymerized with derivatives of adamantane and acrylamide and molded into functional 3D constructs to deliver therapeutic cells (MSC), growth factors (TGF-β1), and other molecules that can help in the regeneration of damaged tissues [56,57]. By incorporation of cationic groups such as ethanolamine functionalized poly(glycidyl methacrylate) to the cyclodextrin host modules, they can also be used as gene vectors as reported by Zhang et al. with good transfection results [58]. Cyclodextrins have also been proven as a delivery system for small molecules such as dexamethasone for regeneration of cartilaginous tissues [59], resveratrol for preventing osteoporosis in postmenopausal women [60], simvastatin to improve water solubility, and osteo differentiation efficiency in bone regenerative therapy [61].

Cucurbit(n)urils (CB[n]) are also a group of “n” number of repetitive macrocyclic glycoluril molecules linked by methylene groups, which possess a hydrophobic cavity such as that of cyclodextrins formed by the carbonyl groups. The number of molecules determines the dimensions of the cavities of these host molecules, where a single guest molecule would require CB(5–7) whereas CB(>8) would be required to host more than 2 guest molecules [62]. As cyclodextrins, cucurbiturils are mostly employed as delivery systems for small and large molecules to the damaged tissues.

Calix(n)arenes are another type of cavitand molecule that form hydrophobic cavities and are named due to their cyclical structural arrangement similar to the form of a Greek Calix-krater vessel [63]. These supramolecular hosts are very flexible with easily modifiable and controllable conformations. Due to this reason, they can be tuned into scaffolds and used as delivery and diagnostic agents.

### 2.3. Natural Biomaterials

#### 2.3.1. Collagen

Collagen is one of the most used biomaterials due to its biocompatibility and being one of the most abundant proteins in the extracellular matrix. Collagen is present in large quantities in bones, skin, tendon, blood vessels, intestine, and cornea. Therefore, it can be used to fabricate diverse types of tissues. The collagen can be either used as the decellularized extracellular matrix or can be used in several types of regenerated collagen biomaterials in the form of hydrogels, scaffolds, or microspheres [64]. Due to its abundance in the bone matrix, collagen is mostly used for cartilaginous tissue and bone regeneration [65,66].

#### 2.3.2. Alginate

Alginate, being a water-soluble polysaccharide, readily forms hydrogels that are biocompatible and biodegradable in the physiological medium. The hydrogels could be formed from the alginate in the presence of bivalent and trivalent cations forming a 3D structure that can carry substantial amounts of water. These hydrogels can carry small molecules, cells, and other delivery systems such as nanoparticles [67]. Martine et al. reviewed the combinations and strategies to make the alginate composites that could promote corneal regeneration [68]. Zhai et al. reported the co-assembled supramolecular structures made up of cell adhesive peptide and the alginate that could promote accelerated wound healing and has haemostatic control [69]. Piras et al. demonstrated a stem cell-compatible dual-network hybrid calcium alginate gel loaded with silver nanoparticles [70].

#### 2.3.3. Gelatin

Gelatin itself is a product of the partial hydrolysis of collagen, which is a major component in the extracellular matrix. Being partially hydrolyzed, it contains a mixture of polypeptide chains. Gelatin also shares the same advantages as collagen by being biocompatible, nonimmunogenic, and being soluble at physiological temperature. Gelatin is also mostly sought out biomaterial used for regenerative medicine, but researchers have been using with synthetic macromolecules to form host–guest interactions that assist in cell filtration. Feng et al. used aromatic residues of gelatin with β-cyclodextrin to form hydrogels that help in cell adhesion and enhance the delivery of hydrophobic drugs. In another study, Zeinab et al. made self-healable biomimetic hydrogel using gelatin, molybdenum disulphide and β-cyclodextrin, which could be used for regeneration of extracellular matrix [71]. Madl et al. prepared the gelatin hydrogels with Cucurbit(8)uril and encapsulated human fibroblast cells, demonstrating its potential application in bioprinting [72].

#### 2.3.4. Hyaluronic Acid

Hyaluronic acid, also a naturally occurring polysaccharide, is also a primary component of the extracellular matrix of the human connective tissue. Hyaluronic acid in physiological conditions undergoes enzymatic degradation in the presence of hyaluronidase and hydrolysis. Hence, hyaluronic acid is made to react with crosslinking agents to form covalent bonds to improve the stability in the physiological conditions. So, HA is used in combination with nucleobases such as cytosine, guanosine to form hydrogen bonding, or combined with Poly(N-isopropylacrylamide) to form hydrophobic bonds making the supramolecular hydrogels. They can form metal co-ordination supramolecular complexes with metal ions such as Mg^2+^, Zn^2+^, Ca^2+^, and Ag^2+^ or also inclusion complexes with Cucurbit(8)urils, adamantane, or β-cyclodextrin [73]. Jung et al. made supramolecular hydrogels that can encapsulate human MSCs and demonstrated chondrogenic differentiation, indicating its use in the cartilage regeneration and other tissue engineering applications [74]. Fernandes et al. prepared supramolecular hydrogels containing HA and cyclodextrin or adamantane for corneal wound healing [75].

## 3. Self-Assembly Mechanism and the Driving Forces

The kinetics of the drug or gene release from the architecture of the supramolecular systems is dependent on the kinetics of the self-assembly mechanism of the systems. Hence, emphasis has been put on the self-assembly mechanism of the various non-covalent bonds that help in the process.

**Figure 4:**
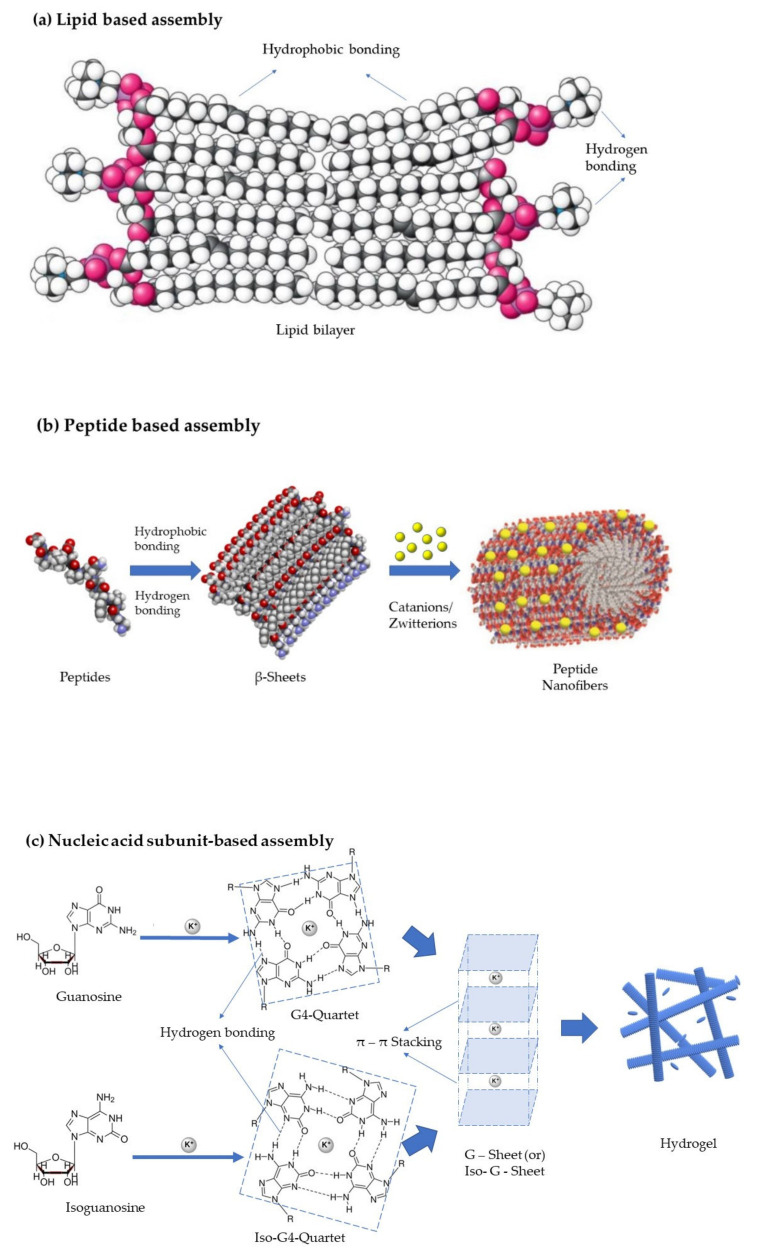
Self-assembly mechanism (created with Biorender.com [accessed on 30 June 2022]).

*Lipids:* The kinetics of lipoplex formation has been critically examined by researchers, and the nucleation and growth processes such as that of the adsorption of the polyelectrolytes on oppositely charged colloidal particles were discovered. Lipid aggregates are amphiphilic and form vesicles or phases/lamellae due to the van der Waals forces between the hydrophobic molecules and the hydrogen bonding between the hydrophilic molecules. When cationic lipids are mixed with negatively charged nucleic acids, rearrangement takes place due to the electrostatic interactions, thus forming multilamellar lipoplexes, as shown in Figure 4a [17].

*Peptides*: Peptide assembly is influenced by noncovalent interactions such as hydrophobic effects, electrostatic interactions, and π–π stacking. Mostly, the amphiphilic peptides that have been used for the self-assembly processes have a surfactant-like structure composed of a hydrophobic tail with many hydrophobic amino acids such as Gly, Ala, Val, Leu, and Ile, and a hydrophilic core represented by one or two hydrophilic amino acids, either negatively charged (Asp or Glu) or positively charged (Lys, His or Arg), resulting in anionic or cationic surfactant-like peptides, respectively. The hydrophobic interaction and hydrogen bonding are the most important driving forces for amphiphilic peptide self-assembly, which lead to the formation of β-sheets that in turn helps in the formation of vesicles or nanofibers. It has also been demonstrated that the addition of an extra ionic bond has a substantial effect on the self-assembling structures. Catanionic and zwitterionic surfactants are the other two forms of surfactants, in addition to typical anionic and cationic surfactants. The former is a mixed system of anionic and cationic surfactants, whilst the latter is made up of surfactants with both positive and negative charges combined in a single hydrophilic head. These materials form lamellar structures, nanotubes, nanovesicles, and micelles, as shown in Figure 4b [22].

Self-assembly peptides that form hydrogels are also widely researched. Hydrogen bonding can occur spontaneously in systems that generate supramolecular fibrils capable of noncovalent cross-linking. Water-soluble fibrils can be made up of either covalently linked polymers or noncovalently linked supramolecular assemblies, with cross-linking occurring via both covalent bonds and noncovalent interactions such as hydrogen bonding, stacking, and van der Waals forces. Braun et al. discovered that the hydrogel-forming peptide SgI37-49 self-assembly is governed by the secondary nucleation of monomers on the surface of pre-existing fibrils. The rate of nucleation in this catalytic nucleation process exhibits enzyme-like saturation effects, with the rate of nucleation being highly concentration-dependent below 50 M and effectively concentration-independent at higher concentrations [76].

*Nucleic acid subunit based self-assemblies*: All the nucleic acid sub-components, namely nucleobases, nucleosides, and nucleotides, serve as supramolecular motifs in the construction of complex architectures via non-covalent interactions such as hydrogen bonding, electrostatic effects, π–π stacking, hydrophobic interactions, and metal coordination. Different structural properties endow them with varying binding abilities. Because of the non-covalent interactions between the components, the resulting assemblies have controllable morphology, physico-chemical properties, and stimuli responsiveness. Commonly used nucleosides for hydrogels are guanosine, Isoguanosine, and ureidopyrimidine, which form quartets by intermolecular hydrogen bonding [77,78]. The molecules in the quartet with the double bonds undergo π–π stacking to form G-Sheets or quadruplexes, as presented in Figure 4c. Such quadruplexes are further grouped to form hydrogels which can be used in regenerative medicine as a delivery vehicle and as a scaffold [79].

*Block co-polymers:* One of the most fundamental driving mechanisms in the supramolecular assembly of tissue engineering complexes is based on hydrophobic interactions. These interactions can cause the association and subsequent self-assembly of supramolecular polymer fibers, or they can produce physical cross-links between polymer chains, which are critical in the self-assembly of biological materials such as fibrillar proteins. There is an increase in the entropy that occurs due to the repulsion of hydrophobic faces away from the hydrophilic environment of the swelling hydrogel and the surface-bound water molecules. This substantial gain in net entropy with a minor amount of enthalpy drives the hydrophobic interactions, which can self-assemble or support the physical structure in the polymer chains. This mechanism is frequently used in conjunction with chemical cross-linking to improve the mechanical properties of the resulting gels, and unlike other supramolecular motifs, it may be used to make robust self-healing hydrogels. Amphiphilic block copolymers that use these hydrophobic bonds to form supramolecular structures are often used for drug delivery, cell encapsulation, and cartilage regeneration. These hydrogel matrices self-assemble in aqueous conditions and dissolve hydrophobic compounds, allowing therapeutic doses of tiny molecules to be delivered locally. In the end, these scaffolds increased cell infiltration, reduced inflammation, and promoted wound healing in vivo, making them one of the few systems whose therapeutic applicability has been proven [80,81,82,83,84].

*Dendrimers:* Amphiphilic supramolecular dendrimers were researched as an alternative for covalently bound dendrimers for biomedical applications. The most widely used dendritic polymers contain small amphiphilic dendrimers containing a hydrophobic alkyl chain and hydrophilic PAMAM dendron. These dendrons can be easily synthesized in large quantities, with high purity, utilizing divergent or convergent methods or a mix of the two. The dendron with both the hydrophilic and the hydrophobic part can self-assemble into stable and resilient noncovalent supramolecular dendrimers. Due to their amphipathic nature, they can have hydrophobic interactions in their core regions, and hydrogen bonds inside their dendron shells. The simplicity to change the length and nature of the hydrophobic chain, as well as the creation of the hydrophilic PAMAM dendron and the varied terminal functions, provides unique structural flexibility and diversity for a diverse range of biological applications [85].

*Host–guest chemistry in cyclic oligomers:* Host–guest interactions in the macrocyclic molecules are one of the most studied non-covalent techniques for forming supramolecular hydrogels. As previously described, most of the cyclic oligomers that are being used for tissue regeneration are cavitands meaning that they have a cavity constructed by the repeating monomer units. In the cyclodextrins, calixarenes, and cucurbiturils, the cavity is hydrophobic due to the aromatic rings and the surface is hydrophilic due to the hydroxyl groups or other hydrophilic moieties protruding at the ends of the cavities. The hydrophobic groups of the monomer units are responsible for the hydrophobic interactions, which are critical in the formation of the host cavity. Guest molecules or the hydrophobically modified polymers are then physically entrapped, forming supramolecular hydrogels. The stimuli-responsive cyclic oligomers contain stimuli-responsive moieties/polymers that initiate the formation of the inclusion complexes without affecting the bulk properties of the oligomers. At the induction of stimuli, these moieties help in the formation of the host–guest interactions. These phenomena were explained well by Saunders et al. by relevant examples [86].

### Natural Biomaterials

*Collagen:* Collagen-based biomaterials, which are majorly fabricated in the form of de-cellularized ECM, have the advantage of less risk of immunological rejection. The collagen is either obtained by decellularization of the organ and recellularized before implantation or is decellularized and stored desperately in distinct biomaterial forms such as gels, for ease of use for in vitro and in vivo applications. This decellularization process preserves the original structure of the collagen, but the major hurdles are to understand the molecular mechanism and to achieve the consistency of the biomaterials during the recellularization process. So, the regenerated collagen biomaterials are often used for the regenerative medicine. One of the most widely used forms of this biomaterial is the hydrogel, prepared by self-assembly process triggered especially by temperature rise to physiological temperatures. To form hydrogels, the collagen solution is mixed with the therapeutic agent and the temperature is adjusted for the physical transition into gels. Another type of collagen-based delivery system is represented by microspheres, prepared by direct aliquoting, or emulsifying the polymer solution, followed by temperature adjustment to form the microspheres and subsequent encapsulation of cells or therapeutic material into the microspheres. Collagen is also used in the form of a scaffold, built three-dimensionally by lyophilization, electrospinning, or 3D printing, to which other cells, genes, or drugs are attached [64].

*Alginate:* Alginate is a biopolymer that readily dissolves in water and forms gels through electrostatic interactions influenced by the electrolytes used. Apart from the gels, alginate has also been used in the preparation of nanofibers, nanocoatings, and nanoparticles, which could be used for therapeutic material delivery. Alginate macromolecules are amphiphilic and generally undergo intra- or intermolecular hydrophobic interactions and hydrogen bonding to form self-assembled nanostructures such as nanoparticles and nanofibers that can carry either drugs or genetic material. Alginate is also used with other supramolecular systems listed earlier in this section such as the cyclic oligomers, peptides, and other natural biomaterials to form these self-assembled systems. Characteristics of alginate, such as the resemblances to the native ECM and ability for permeation of gases and small molecules through it when prepared in the form of tissue-engineered scaffolds are the major reasons that it has been extensively researched for potential in tissue regeneration and wound healing [87].

*Gelatin:* Generally, gelatin does not form supramolecular systems on its own and therefore it is used in combination with other supramolecular systems. Cross-linking is generally carried out in the presence of chemical reagents that form covalent bonds or by the dehydrothermal method that uses vacuum heating for the formation of gelatin microparticles. These systems have been used for various regenerative medicine applications reviewed by Nii et al. [88]. There are studies that use these biomaterials with cyclic oligomers or other reagents employing host–guest chemistry to form inclusion complexes and other supramolecular structures [71,72,89,90,91].

*Hyaluronic acid:* Hyaluronic acid, which is hydrophilic and can be easily chemically modified, generally forms supramolecular hydrogels which are used as scaffolds for tissue engineering and regenerative medicine. Hyaluronic acid readily forms hydrogen bonding to form these reversible hydrogels, but the interactions in water or aqueous solution weaken the bong. Hence, HA is often combined with materials such as poly(N-isopropylacrylamide) (PNIPAM) to form hydrophobic interactions and to obtain stronger hydrogels. Possessing negatively charged carboxyl groups at physiological pH, HA can interact electrostatically with positively charged groups such as carboxymethyl hexanyl chitosan or chondroitin sulfate. It is also reported that HA forms coordination bonds with bivalent and trivalent metal ions such as the Mg2+, Cu2+, Fe2+, Ba2+, and Ca2+. Recently, the most extensively researched hydrogels have been those based on HA with cyclic oligomers such as β-CD or Cucurbiturils, which use the host–guest chemistry to form the inclusion complexes [73].

## 4. Preparation Techniques and Formulation Considerations

### 4.1. Lipid-Based Systems

**Figure 5:**
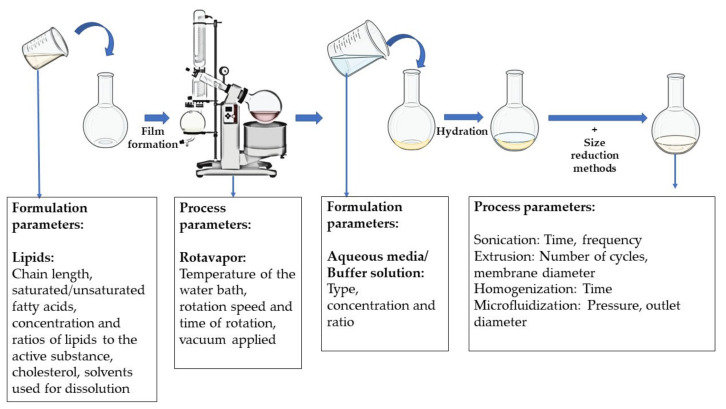
Schematic representation of the preparation of liposomes, highlighting the formulation and process parameters involved (created with Biorender.com [accessed on 30 June 2022]).

When lipids are immersed in an aqueous environment, their amphiphilic nature allows them to form structured assemblies such as vesicles or membranes. Lipid bi-layer sheets and liposomes are being researched majorly as a delivery vehicle for the small and large molecules rather than a biomimetic scaffold. In regenerative medicine, they have been applied to deliver the stem cells with growth/differentiation factors, DNA, or interference RNA (RNAi) and in combination with scaffolds. Preparation of liposomes can be performed by a conventional method of thin-film hydration, and then loading is performed actively or passively with small drug molecules or biologically active macromolecules. The passive loading mechanisms such as the mechanical dispersion method, solvent dispersion method, and detergent removal method are commonly followed. There are many types of mechanical dispersion such as French press extrusion, freeze-thaw, micro-emulsification, membrane extrusion, and others [92]. Many new techniques have been employed lately, which were reviewed by Has et al., for both hydration and loading techniques. Modified methods include electro formation, microfluidics-assisted methods, curvature tuning method, packed bed-assisted hydration, osmotic shock method, dual asymmetric centrifugation, spray-drying, and lyophilization [93]. The schematic representation of the preparation of liposomes is given in Figure 5.

In the conventional hydration techniques, usually, the essential materials are lipids, active substances, and the buffer solution. The critical quality attributes of the liposomes are majorly affected by the physiochemical properties of these components. Porfire et al. discussed the critical material and process attributes that influence the liposome properties. They discussed that saturated fatty acids have a higher transition temperature and do not undergo oxidative or hydrolytic degradation such as unsaturated fatty acids. The chain length of the lipids will influence the thickness of the lipid bilayer which is inversely proportional to the internal vesicular volume for encapsulation of the therapeutic molecules. The lipids also impact the fluidity and permeability of the bilayer and the charge. Cholesterol used usually for mechanically supporting the lipid bilayer also impacts the encapsulation efficiency. The solvents used, the concentrations and ratios of lipids, solvents, and active substances have a profound effect on the target profile of the liposomes [94].

In addition, many material attributes vary according to the type of preparation process selected. For the thin-film hydration technique, the temperature of the water bath, the speed and time of rotation of the flask, and the vacuum applied might influence the liposome size, but it is the type of the size reduction methods employed that has a greater impact on the final properties of the liposomes. For example, if the liposomes are extruded for size reduction, the diameter of the extrusion membrane and the number of cycles influence the particle size of the liposomes.

### 4.2. Peptide-Based Systems

Peptide nanofibers and hydrogels have been used in regenerative medicine due to their ability to exhibit bioactive signals, which can be performed by integrating the bioactive component within the peptide sequence or co-assembling the peptide with the bioactive signal. Hence, these two types of delivery systems are much more focused on by the scientists to improve their applicability as not only an extracellular matrix scaffold but also as a delivery vehicle for small and large molecules in the biomedical domain. Peptides and peptide amphiphiles, as discussed earlier, form multiple conformational structures such as micelles, β-sheets, and nanofibers when influenced by the external environment subjected to changes in pH, temperature, and electrolyte ions. Nanofibers have been the most applicable form in tissue engineering. There are various methods of manufacturing nanofibers such as template synthesis, phase separation, melt-blown technology, force spinning, and freeze-drying, but electrospinning is mostly preferred for peptide nanofibers [95].

**Figure 6:**
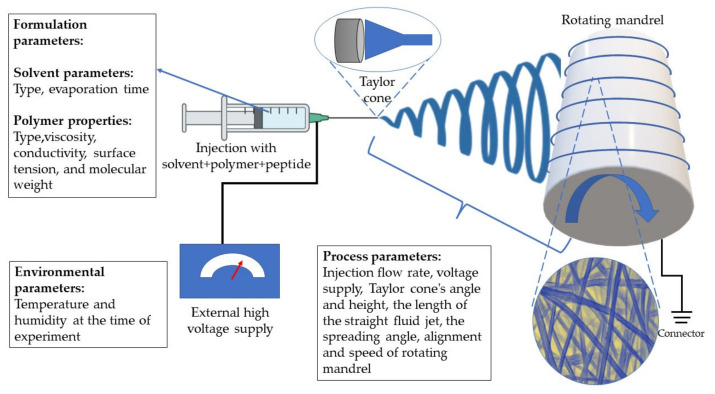
Pictorial representation of electrospinning process.

Electrospinning is a low-cost and easily accessible method for producing nanofibers using electrical forces and polymer solutions, in a short amount of time with surface control of nanofibers [96]. A spinneret, a high voltage power supply, and a grounded collecting plate are the three fundamental components of an electrospinning apparatus (usually a metal screen, plate, or rotating mandrel). The principle of this technology is to apply high voltage to a polymer solution to generate electrical field jets, and then evaporate the solvent while the formed jets flow to a grounded collector to obtain nanofibers. The process is diagrammatically represented in Figure 6.

Parameters related to the polymer solution, process parameters, and environmental conditions affect the electrospinning process. The viscosity, conductivity, surface tension, and molecular weight of the polymer are all formulation factors that have a substantial impact on the morphology and size of the fibers. The process parameters are the applied electrical force, tip to distance collector, and feed/flow rate. The electrospinning process is also influenced by environmental circumstances, which include the temperature and humidity at which the operation is carried out, to avoid degradation of the peptides. In addition to these parameters, the formulation additives such as the type of organic solvent used to dissolve the peptides and to enhance the spinnability. To improve mechanical qualities, peptide/proteins can be combined with natural and synthetic polymers such as Eudragit^®^, poly-L-lactic acid (PLLA), polycaprolactone (PCL), chitosan, polylactic acid (PLA), and PEO. Morphology, fiber stability, decreased degradation in the physiological environment, increased biological function, and increased spinnability are examples of these features. Glutaraldehyde, formaldehyde, genipin, proanthocyanin, and chlorination are examples of cross-linkers that can be utilized for this purpose [95].

### 4.3. Nucleic Acid Subunit-Based Systems

Nucleosides such as guanosine and Isoguanosine assemble in quartet structures to form hydrogels which can be used for regenerative medicine. The nucleobases associate through Hoogsteen hydrogen bonding to form vertical stacks of square-shaped quartet aromatic structures and grow into wires or fibrous structures which drive the gelation process. These self-assembled hydrogels are either stabilized by cations or anions. Many different preparation methods have been reported by researchers. The common main components are the gelator molecules, which are the nucleosides such as guanosine or Isoguanosine in their stable forms such as hydrazides or aldehydes which, in the presence of monovalent or bi-valent cations and anions, yield self-assembling structures [77].

### 4.4. Block Copolymers (BCP)

Amphiphilic block copolymers majorly form three types of assembled structures, for example micelles, polymerosomes, and nanoparticles. Micelles are self-assembled nanostructures formed when the concentration of the BCP in dilute solutions is above the critical micellar concentration (CMC). These micelles serve as excellent carrier systems for drugs and biomolecules. Polymerosomes are also self-assembled structures and differ from the micelles by being double-layered vesicular structures [97,98,99].

### 4.5. Dendrimers

**Figure 7:**
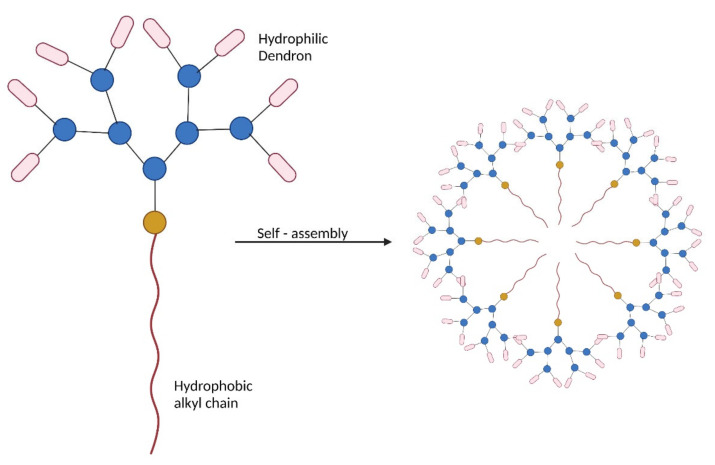
Amphiphilic dendritic copolymers (created with Biorender.com [accessed on 30 June 2022]).

The dendrimers are either prepared by convergent or divergent methods consisting of a multifunctional central core with branching dendritic polymers involving the formation of covalent bonds. Due to this covalent synthesis, the higher generation of dendrimers, which have many branches, often has structural defects due to incomplete reactions. In the case of amphiphilic dendritic polymers, dendrons are also manufactured either by convergent or divergent methods. However, then the hydrophilic dendrons have hydrophobic alkyl chains that form the core by electrostatic and hydrophobic interactions. This problem could be overcome by the non-covalent synthesis using the amphiphilic dendritic polymers with hydrophobic alkyl chains and the hydrophilic dendrons consisting of PAMAM. The hydrophilic dendrons are directed towards the outer surface, while the hydrophobic alkyl chain is directed towards the center, as shown in Figure 7. The hydrophobic alkyl chains from various dendrons form the core by electrostatic and hydrophobic interactions [44].

Chen et al. synthesized cationic dendrimers with hydrophilic PAMAM dendrons with seven tertiary amines in the inner structure and eight of them on the terminals using click chemistry with triethanolamine as the core. They observed that the longer alkyl chains improve the hydrophobic interaction and the stability of the complex. Additionally, the dendrimers with fewer generations or shorter alkyl chains led to insufficient genetic material loading. This might be due to the lesser hydrophobic groups when compared with the longer alkyl chains, which impacts the electrostatic interaction with the genetic material [100].

### 4.6. Cyclic Oligomers

Cyclic oligomers being used in regenerative medicine are synthesized mostly chemically, and then the inclusion complexes are formed through various preparation methods to host the guest molecules of drugs and other biomacromolecules. Cyclodextrins are prepared by the enzymatic degradation of starch by using glucosyltransferase.

The preparation of inclusion complexes of cyclic oligomers is critical in the performance, morphology, and stability of the delivery systems. The inclusion complexes are prepared by many methods, namely co-precipitation methods, kneading, grinding, spray drying, irradiating by microwaves, and by the utilization of super critical fluids [101]. Co-precipitation is one of the most simplistic and efficient methods where the guest molecule to be hosted is dissolved in organic solvents to which solution containing the cyclic oligomer is added and agitated. The solution is then cooled until crystallization occurs. The crystals are then washed with organic solvents and dried [102,103,104]. A simple method called the kneading method consists of making a paste of cyclodextrins with a small amount of water and adding the guest molecules with thorough mixing. Then the resultant paste is washed off with solvents to obtain the solid form of the inclusion complex [105]. An alternative method to the above two techniques is the utilization of super critical fluids. Most commonly, super critical carbon dioxide is used, which is maintained at super critical conditions of temperature and pressure in an autoclave, with the cyclodextrins and the guest molecules. A rapid pressure drop occurs due to which carbon dioxide is then vaporized, and the inclusion complex is formed. A more simplistic method is the grinding method, which involves mechanical energy that gives sufficient intensity to the guest molecules to become trapped in the host molecules. Some methods employ microwaves to blend guest molecules and the host molecules in a minimum amount of solvent such as ethanol [106,107]. For water-soluble or water-dispersible guest and host molecules, spray drying could be utilized to form the inclusion complexes. In this technique, the liquid containing the molecules is atomized into fine droplets which are then dried by a heated air stream and separated from the air.

## 5. Characterization

Supramolecular-based systems usually involve self-assembling units (both biomacromolecule based as well as synthetic) whose equilibrium constant varies with the varying chain lengths. For such dynamic systems, a single characterization technique might often not be sufficient, and a combination of techniques would be required to evaluate them qualitatively and quantitatively [108]. Additionally, these systems are mostly produced in the form of gels that mimics the extracellular matrix of the target tissue or act as delivery systems to carry therapeutic material to the targeted site. So, the characterization mostly involves similar testing methods as that of viscous gels. The general characterization of other delivery systems would also be taken into consideration, but majorly the supramolecular mechanism was studied and represented in Table 1.

### 5.1. Rheology

Rheology in practical terms can be defined as the study of the flow and deformation of materials under the applied external forces [109]. It is primarily focused on relating the force with the deformation in materials such as liquids and semisolids, which in turn can provide valuable information about dynamics and structural assemblies. The researchers test the elastic storage modulus (G’), the measure of energy stored during a strain cycle, and elastic loss modulus (G”), the measure of energy lost during a strain cycle, to assess the nature of the supramolecular structure and assemblies. Dawn et al. detailed frequency sweep experiments, and time-dependent experiments, about the determination of yield stress and linear viscoelastic region. According to the G’ and G” the supramolecular gels were categorized into healable and non-healable. Further healable systems were sub-categorized into fibrous, non-fibrous, self-healing, and stereochemistry-based systems based on the type of framework [110].

### 5.2. Chromatography Techniques

Chromatographic techniques such as size exclusion chromatography have often been used for polymer characterization and to provide information about the molecular mass distribution of the polymers. Gel permeation chromatography was reviewed by Liu et al., where they considered research on supramolecular systems such as dendrimers with multiple hydrogen-bonding arrays [108]. These techniques, when combined with light scattering and x-ray scattering techniques, will provide the average molecular weight of the polymer.

### 5.3. Spectroscopy Techniques

Based on the information sought regarding self-assembled polymer structures, a variety of spectroscopic techniques are available for characterization. All the spectroscopic techniques used for supramolecular systems are reviewed in this section.

Infrared spectroscopy could be used to characterize the non-covalent interactions behind the gelling process of supramolecular systems. IR spectroscopy can be used to explain the formation of intermolecular hydrogen bonding during aggregation, and the formation of new bands in the CH stretching areas because of the packing of aliphatic chain molecules [111].

Ultraviolet and visible light is often absorbed by molecules to excite the electrons from the π bonds or the nonbonding electrons to higher antibonding molecular orbitals. In supramolecular systems, non-covalent interactions can also cause physical changes, for example, in the case of gelation, the interactions increase the hydrophobicity of the surroundings [112,113]. In the case of stimuli-responsive supramolecular systems, where physical changes are influenced by external stimuli, UV/Visible spectroscopy can be used to quantitatively determine the non-covalent interactions [53].

Fluorescence spectroscopy is another type of electromagnetic spectroscopy that is used for the quantitative determination of supramolecular systems. The principle differs from the UV/Vis by analyzing the fluorescence of the electrons that are previously excited and lose their vibrational energy to come to the ground state [114,115]. It can also provide details regarding the inclusion of complexation by cyclic oligomers [116].

^1^H NMR probes hydrogen nuclei within the molecules of a substance to determine the structure and the interactions of its molecules. In the supramolecular systems, the chemical shift changes that are driven by noncovalent interactions can be monitored [117]. Diffusion-ordered NMR spectroscopy (DOSY) has become significant in the characterization of self-assembly systems for being able to measure translational diffusion coefficient that accounts for the net result of thermal motion induced in solution by particles or molecules [118,119]. This coefficient can provide accurate hydrodynamic dimensions of the systems by using the Stokes–Einstein equation as well as the thermodynamic parameters. Nuclear Over Hauser effect spectroscopy (NOESY) [56,120] and rotating frame nuclear Over Hauser effect spectroscopy (ROESY) [121,122,123] have also been used to obtain information about the relative positions of the building components in host–guest interactions of cyclic oligomers by employing two-dimensional NMR.

Mass spectrometry has been employed in some situations where chemical composition and end group identification for supramolecular polymers had to be determined [111,124,125,126].

Circular dichroism spectroscopy is a technique that involves the study of the stereo structures and the intra- and intermolecular interactions of various classes of chiral supramolecules. This technique always is used to monitor the thermal responsive changes in nanoscale chiral aggregates assemblies [115,126].

### 5.4. Dynamic Light Scattering and X-ray Scattering Techniques

The dynamic light scattering technique (DLS) is used to determine the size distribution profile, structural formation, and interactions of small particles in suspension or small polymers in the solution. When a particle is irradiated and subjected to Brownian motion, two frequencies of equal intensity, including a positive and negative Doppler shift proportionate to the particle velocity, are generated in addition to the frequency that would usually be scattered. Brownian motion causes interference between the non-shifted wave (proton re-emission) and the two waves, resulting in microscopic intensity changes. The scattered intensity is measured as a function of time and self-correlated after that. The relaxation time due to Brownian motion is obtained, and the particle size is determined using hydrodynamic models of the diffusion coefficients. The diffusion coefficient can also be calculated using DLS, which simply monitors changes in the scattered light intensity of diffusing particles. The study of light scattering by structures with diameters in the sub-micrometer range allows key features such as shape and internal structure to be determined. Because the size (or diffusion coefficient) increases drastically when 3D networks grow, DLS is an effective tool for the characterization of supramolecular gels [127,128,129,131].

X-ray techniques have been transformed significantly over the past two decades to cater for the characterization of structural motifs of the supramolecular assemblies and to understand the weak intermolecular interactions and the local packing of the molecules. To determine the arrangement of atoms, a beam of X-rays impacts the material and causes the beam of light to spread out in many different directions. A crystallographer can create a three-dimensional picture of the density of electrons within the system by measuring the angles and intensities of these diffracted beams. The mean locations of the atoms in the system, as well as their chemical bonds, disorder, and other information, may be deduced using this electron density [77]. This X-ray diffraction method was employed by Hwang et al. in the characterization of cucurbiturils to determine the layer structure and the alignment of the fibrils during self-assembly [132]. Angelerou et al. used this technique in the characterization of nucleoside-based supramolecular gel to check the local packing of the molecules in N4-octanoyl-2′-deoxycytidine xerogels [125].

### 5.5. Thermal Analysis

In supramolecular chemistry, noncovalent interactions involve energy and momentum transfers between molecules. Isothermal titration calorimetry can be used to determine these thermodynamic parameters such as binding affinity, enthalpy changes, and the stoichiometry of the interaction between the molecules. Differential scanning calorimetry (DSC) is a thermoanalytical technique that measures the difference in the amount of heat required to raise the temperature of a sample and a reference as a function of temperature. These techniques can not only determine the sol–gel transition enthalpy but also the thermal stability of the supramolecular gel. Xia et al. obtained enthalpy changes and the thermal stability of the oligopeptide hybrid films using DSC [133]. Park et al., used this technique to explain the reversible phase transition of their β-cyclodextrin-based noncovalent, double-network hydrogel [130].

### 5.6. Microscopic Techniques

Microscopy techniques are mostly used together with other characterization techniques and often a combination of microscopic techniques is used to understand the morphology of the sample. Scanning electron microscopy (SEM) is a type of electron microscopy that uses a focused beam of electrons to scan across the material to create images. These electrons interact with the electrons in the sample, resulting in a variety of signals that may be detected and carry information on the surface topography and composition of the sample [111,134,135]. In most cases, the electron beam is scanned in a raster scan pattern, and the position of the beam is coupled with the detected signal to create a picture. Another microscopy technique is transmission electron microscopy (TEM), in which a beam of electrons interacts with the object as it goes through. The interaction of the electrons with the specimen can result in the formation of an image. An imaging device, such as a fluorescent screen, a layer of photographic film, or a sensor, is used to magnify and focus the image (CCD camera). In theory, TEM has enough resolution to detect molecules at the sub-nanometer scale [111,136,137]. One of the most important tools for photographing, measuring, and manipulating materials at the nanoscale is the atomic force microscope (AFM). The data are collected by ‘‘feeling” the surface with an extremely sensitive ‘‘spring-board-like” cantilever. Attractive or repulsive interactions that influence the tip at the end of the cantilever as it is moved over a sample cause the cantilever to bend, thereby providing a mechanical means to probe local nanoscale effects [77,118,136]. These techniques have been used widely to study the structural and morphological details of the materials. In the supramolecular systems, the individual fibers that self-assemble to form the hydrogels could be visualized with higher levels of a resolution, allowing the user to determine the structural changes with and without the therapeutic molecules. This electron microscopy when coupled with a focus ion beam allows 3D imaging of the supramolecular systems. Cryo-electron microscopy techniques step up in providing the observations of the hydrogels at different gelator concentrations and observing the nano-structural changes during their synthesis.

## 6. In Vitro and In Vivo Biocompatibility Studies

**Figure 8:**
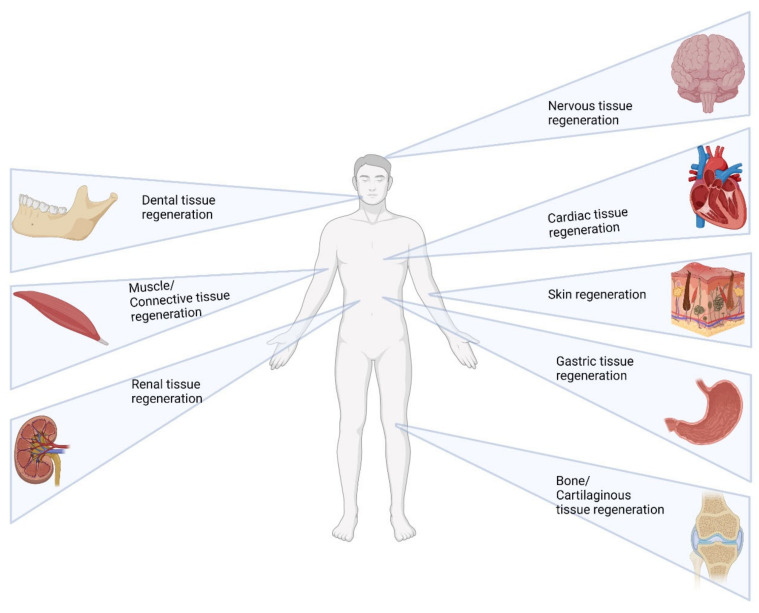
Targeted tissues for regeneration using supramolecular systems (created with Biorender.com [accessed on 30 June 2022]).

One of the most important characteristics of any delivery system is its biocompatibility and, since it is being employed in regenerative medicine, the supramolecular systems need to be more bio-compliant than other delivery systems. Most of the prototypes successful in the in vitro studies usually become phased out due to their inability to prove their efficacy in in vivo models. Hence, a major emphasis has been given to the successful prototypes and research activities held previously, proving the biocompatibility of both the biomacromolecule-based and synthetic macromolecule-based supramolecular systems. Some of the most recent development and studies regarding the biocompatibility of the supramolecular systems with the various tissues are tabulated in Table 2 and Figure 8.

## 7. Innovations and Future Approaches to Improving Clinical Applicability

### 7.1. Stimuli Responsivity—Control Using Various Factors Such as pH, Temperature, Light, Enzymes and Others

Despite enormous research in drug research and development, more than half of potential treatments fail in clinical trials due to a lack of therapeutic efficacy and unacceptable safety. The therapeutic effectiveness and safety of a pharmacological agent are heavily influenced by drug formulation and delivery techniques. The use of stimuli-responsive triggers to modulate drug biodistribution such that a drug acts both when it is needed and at the place where it is needed is one commonly researched way toward more efficient delivery systems. Supramolecular systems can be designed to accompany stimuli-responsive moieties that can define their mechanism of action and the kinetics in the human body. When external stimuli such as changes in pH, temperature, light, electrical activity, or enzymes are detected, these stimuli-responsive systems change their shape or volume which causes them to grow or decrease in volume and consequently release the therapeutically active substance [165].

Alternatively, the process might be made more restricted by incorporating the ability to respond to pathophysiologic markers, such as changes in blood pressure, pH, an increase in redox activity, or a rise in enzyme concentrations. Injectable hydrogels that are responsive to pH, temperature, and enzymes have been studied for site-specific controlled drug delivery since any illness in the body induces conditions of low pH, increased temperature, and varying glucose concentrations. For targeted delivery systems, light and enzyme-responsive hydrogels are preferred, whereas pH-, temperature-, electric-, and light-responsive hydrogels can be employed for controlled release delivery systems [166].

#### 7.1.1. Temperature Responsive Systems

Temperature-responsive supramolecular hydrogels are the most researched stimuli responsive systems in the supramolecular systems due to their applicability. They undergo sol–gel or gel–sol transitions in response to minor changes in their surrounding temperature. The temperature-dependent phase changes of supramolecular hydrogels are predominantly driven by hydrophilic and hydrophobic interactions, like thermoreversible hydrogels. Biomaterials that display a sol–gel transition at physiological temperatures (37 °C) are ideal for use as scaffolds and for delivering biomolecules in tissue engineering [167]. Hao et al. designed a flexible self-assembling system formed from the complexation of a long-chain amino amide and a trifunctional carboxylic acid. This supramolecular system forms a reversible thermo-responsive gel with the sol–gel transition ranging between 23 and 76 °C [168]. In another example, self-assembling biomimetic β-sheet rich peptides when complexed with polysaccharides such as guar gum resulted in a thermoresponsive hydrogel that undergoes the sol–gel transition at variable temperatures [169]. A self-healing gel made from the ABA triblock copolymer was prepared by Wang et al. for postoperative tissue regeneration for gastric perforation [151].

#### 7.1.2. Light Responsive Systems

The employment of hydrogelators encoded with light-sensitive groups (e.g., photocleavable groups, photo-isomerizable groups, photo-dimerizable groups) to cause phase transitions is another strategy to design the stimuli-responsive systems [170,171]. Light-induced cis-trans isomerization is also termed photo isomerization and involves groups such as azobenzene and spiropyran. Zhao et al. used photo-responsive supramolecular hydrogels made up of azobenzene and α-cyclodextrin groups attached to hyaluronic acid chains, forming host–guest interactions. The azobenzene undergoes cis-trans isomerization upon application of the UV light and the epidermal growth factor (EGF) is released at the wound site for accelerated wound healing [53]. Photocleavable hydrogels employ o-nitrobenzyl, coumarin, or other groups which could be easily dissociated with exposure to UV light. Upon cleavage, the active biomacromolecules or drugs can be released from the hydrogels [172]. There are also photodimerizable groups, which have been used to make these photo-responsive systems, which could be either crosslinked or de-crosslinked, and readily transition to gel or sol upon light irradiation. Coumarin, cinnamic acid, and anthracene are some of the groups that exhibit photodimerization [173].

#### 7.1.3. Electric and Magnetic Responsive Systems

Hydrogelators containing magnetically active cores, such as paramagnetic or diamagnetic species (e.g., metal ions, aromatic moieties) that form aligned fibrillar structures in the presence of an external magnetic field, are used to create magnetic field-responsive supramolecular hydrogels. In most cases, magnetic nanoparticles are incorporated into the hydrogel matrix to produce these magnetic hydrogels. Zhang et al. developed a system based on biocompatible supramolecular polymeric nanofibers. They developed magnetic nanoparticles modified with actin-binding peptide, adamantane (MS-ABPAda), and cyclodextrin-bearing hyaluronic acid host polymer (HACD) that could remodel the stem cell polarization and could be applicable for tissue regeneration [54]. Another example is the inclusion of magnetic nanoparticles in peptide hydrogels made up of Fmoc-diphenylalanine and Fmoc−arginine−glycine−aspartic acid (Fmoc-RGD) short peptides which were tested in vivo in mice and were biocompatible and biodegradable [174]. Another category of stimuli-responsive hydrogels which are closely linked to magnetic responsive hydrogels are the electro-sensitive supramolecular hydrogels based on compounds such as (poly(3,4-ethylene dioxythiophene) polystyrene sulfonate), 3,4-dihydroxyphenylalanine (DOPA), diketopyrrolopyrrole which are incorporated into peptide-based hydrogels. These systems are applied in biomedical fields such as biosensing, drug delivery, and tissue engineering domains [175].

#### 7.1.4. Chemical/pH-Responsive Systems

The use of pH as a stimulus is one of the most effective ways to start supramolecular hydrogelation since a small amount of acid or base can cause a substantial pH shift by fast diffusion of protons or hydroxide ions. Because biological tissues have varying pH levels (gastric pH of 1–3 vs. intestinal pH of 6.1–7), pH-responsive supramolecular hydrogels can be employed as smart drug delivery vehicles. In addition, several diseased diseases, such as cancer, have been shown to have a different extracellular pH than the healthy state. The transport of active substances has often been aided by the difference in pH within the damaged tissues. Ghosh et al. prepared supramolecular hydrogel based on PEP-1, which is an octapeptide containing pH-responsive Aspartic acid and Leucine residues, which could host water-soluble guest molecules. These residues were responsible for the disassembly of the structure of the peptides in the acidic pH, thereby releasing the guest molecule into the vicinity [176]. Similar, hydrogels were made from peptides whose structural assembly can be modified with the change in the pH capable of forming an extracellular matrix with selective cell proliferation and cell adhesion ideal for tissue regeneration [177,178].

Another way to make supramolecular hydrogels is to use certain chemical compounds as a trigger for self-assembly. The use of specific small molecules as a trigger to drive/disrupt self-assembly is difficult because it requires strong molecular recognition to ensure efficacy and avoids nontarget effects. For example, reactive oxygen species (ROS), which are a major cause of oxidative stress and are associated with a range of diseases such as cancer and intervertebral disc degeneration, can be used as a trigger to release the drugs. Functional groups such as ferrocenyl groups when attached to the supramolecular systems can respond to the redox changes in the system [179]. Milcovich et al. fabricated ECM-inspired hybrid microspheres that are made up of collagen 1 and polypropylene sulfide (PPS), which exhibited high responsiveness to ROS, and that could be used as a platform to deliver drugs and other biomolecules [180]. The release of doxorubicin has been demonstrated by Zuo et al. from the star-shaped amphiphilic co-polymers with β-cyclodextrin and ferrocene pair which are dissociated due to the oxidation caused by ROS [181].

#### 7.1.5. Biological/Enzyme Responsive Systems

Biological responses and enzymatic reactions play a crucial part in the creation of hierarchical structures in nature. The supramolecular systems that are influenced by these biological stimuli and enzymes have gained a lot of interest in recent years due to their inherent flexibility to easily undergo structural or phase transition changes in-situ, thereby releasing the genes or drugs at the target organ [182,183].

There are many research publications citing this enzyme responsivity, one of the first one being from Vemula et al.’s group, who prepared supramolecular hydrogels with curcumin encapsulated in them. Using enzymes such as hydrolase, they demonstrated the release of curcumin from the hydrogels [184]. Unlike the former group, Yang et al. made supramolecular hydrogels from pentapeptides hydrogelator, Nap−FFGEY attached with enzymes such as kinase or phosphatase, which can induce a sol–gel transition. In the presence of the adenosine triphosphates and kinase, the gel is transitioned into sol due to the conversion of tyrosine residue into a relatively hydrophilic tyrosine phosphate. The sol–gel transition is triggered when the phosphatase enzyme is added, which counteracts the former mechanism by making it more hydrophobic [184]. Yu et al. used polymeric cyclodextrin and acrylamide or N-vinyl pyrrolidinone to form a host–guest supramolecular hydrogel. The guest molecules with two adamantane moieties form crosslinking between the hydrophobic cavities of the cyclodextrin and the β-lactam core, which could be broken down in the presence of the β-lactamases enzymes. This phenomenon degrades the hydrogel, thereby releasing drugs or biomolecules [185].

#### 7.1.6. Multi-Stimuli Systems

Most biological systems can respond to a variety of stimuli. Supramolecular hydrogels with multi-stimuli responsiveness, including pH, temperature, light, enzymes, redox potential, electric or magnetic field, or tiny molecules, have received a lot of attention. These multi-stimuli-responsive hydrogels respond to two or more stimuli in a sequential or concerted manner and can be used to control phase transition behavior. By employing precursors that are responsive to multiple stimuli and harnessing host–guest interactions to generate supramolecular hydrogels, it is possible to create multi-stimuli-responsive systems. Supramolecular dendronized copolymers were prepared by Zou et al. that could be regulated by the pH and the temperature for applications in drug delivery and tissue engineering [186].

### 7.2. Molecular Simulations

The design of supramolecular systems was mostly carried out by trial and error, and now the researchers have been trying to find quantitative (molecular) structure–property relationships (QSPR) that would be useful in cases such as protein-drug binding. The spatial resolution of experimental techniques is insufficient to provide information on the exact conformation of the individual building blocks. In most cases, this means that the packing of molecules can only be explained by combining a variety of experimental techniques. The fact that the nanostructures frequently are dispersed in their solvent excludes the use of common structural determination techniques such as X-ray crystallography or solution-phase NMR. Hence, a variety of techniques such as light absorption (UV/Vis, infrared (IR) and circular dichroism) and scattering (dynamic and static light scattering (DLS/SLS)), wide or small-angle X-ray scattering (WAXS/SAXS), and small-angle neutron scattering (SANS) are needed to be applied to gather small molecular conformations information to determine the intermolecular interactions. As most of these techniques are time-consuming, measuring the averages and using empirical models developed to mimic the macromolecular systems, there are high chances that the potentially important information is missed, or false interpretations are made. Using classical molecular simulations is one way to overcome these barriers. This computational method follows the movement of individual atoms or molecules over time, eliminating the need for ensemble averaging and mechanistic limitations [187].

Molecular simulations help us to create new self-assembling biomolecular systems using in silico methods and directly validate spectroscopic or scattering results by directly linking molecular simulation output with experimental observations. The molecular dynamics technique (MD) can be employed to study the supramolecular self-assembly by non-covalent interactions. All atoms (AA) are treated as interaction centers in MD and related techniques, in which force fields such as AMBER, CHARMM, OPLS, and GROMOS are applied, which can specify the configuration of the supramolecular aggregate and can be calculated by quantum mechanics. Such techniques give us information about the packing motifs, self-assembly pathways, and starter molecules.

However, all-atom models are frequently insufficient for reproducing crystal packing and melting data, inefficient pH models, extensive sampling techniques, and the requirement of large computing resources. In such situations, coarse-grain (CG) force fields, which connect groups of atoms into effective interaction centers, are employed. These force field techniques can address the large-scale self-assembly and can execute high-throughput simulations. Often, both these force fields are compared and calibrated using the regular analytical techniques such as microscopy, scattering techniques, NMR, and Electron paramagnetic resonance (EPR) spectroscopies. They are also indirectly compared for certain quantitative measurements such as the peptide secondary structure through IR spectroscopy and electronic structure through UV/Vis spectroscopy [187].

### 7.3. 3-D Printing

3D printing is a rapidly evolving manufacturing technology that has the potential to create 3D objects by depositing multiple layers of material. It was previously thought to be primarily useful for prototyping, but recent advances in this field indicate that it is now emerging as a disruptive technology capable of large-scale production scenarios. In regenerative medicine, this technique can be instrumental in providing the solution for scaffolds that can be structurally designed with specificity to location and biocompatible by employing inks made up of safe biodegradable polymers. Owing to their tunable viscosities and reversible mechanical properties, supramolecular polymers are ideal candidates for use in hybrid jetting inks.

Moroni et al., in their review, described the biofabrication strategies that can be employed to construct in vitro 3D tissue models from the mixture of biomaterials and cells. They described that the supramolecular biomaterials are ideal bioinks for to their ability to undergo shear thinning during flow due to the physical non-covalent bonds. Materials such as β-cyclodextrin in modified hyaluronic acid polymers could be broken down during extrusion and rapidly stabilized when deposited. Genetic material such as DNA also can be used in combination with polypeptides, forming a supramolecular assembly that can be easily broken down by the proteases and the nucleases. Natural biomaterials such as alginate also can act as bases onto which the complimentary peptides can be grafted, which protects the cells from external pressure during the extrusion.

Such hydrogel-forming systems are utilized to form the in vitro 3D tissue models that could also be used to check the safety and efficacy of the therapeutic agents. These models are mostly prepared as the cellular spheroids or the cell-laden hydrogel constructs that mimic the native tissues structurally and functionally. For example, to prepare a skin model, a hydrogel containing layers of melanocytes and keratinocytes could be considered. This skin model could be used to study the safety of the chemical compounds. A bioink consisting of human induced pluripotent stem cells can be utilized for making a liver model and can be used for evaluating metabolic activities [188]. In addition to the tissue models, researchers have been making 3D culture systems which are inducted biologically with the ECM components such as chitosan, alginate, collagen, gelatin, or hyaluronic acid. These models are being used for drug screening in the cases of disorders and diseases such as cancers. The stromal cells such as fibroblasts, macrophages, mesenchymal stem cells, and endothelial cells are co-cultured with the cancer cells to create the 3D models, to create a more realistic tumor environment [189]. For example, to investigate tumor-targeting drugs for neuroblastoma, an in-vitro 3D model was created by Kock et al. by co-culturing neuroblastoma and fibroblast cell spheroids [190]. Similarly, a novel scaffold-free 3D model was developed by a combination of pancreatic cells (PANC-1), endothelial cells, and fibroblasts by Lazzari et al. They managed to combine the tumor and stromal components for creating tumor spheroids for preclinical screening of drugs for pancreatic cancer [191].

Hart et al. described the use of a biodegradable poly(caprolactone) diol [M_n_ = 2000 Da] that was added to a series of hydrogen-bonding moieties, resulting in a supramolecular polymer with good solubility. A self-supporting twisted pyramid was also printed to show that the method could be used to deposit more detailed structures. The polymers’ biocompatibility was also investigated, and cytotoxicity tests revealed that they were nontoxic following ISO 10993-5 and 10993-12. Cell attachment was also investigated, and confocal microscopy confirmed that the addition of hydrogen-bonding motifs to the biocompatible poly(caprolactone) did not affect cell attachment [192]. Several factors govern the printing process; an assessment has been carried out by Sather et al. to find what factors can contribute to the printability of the liquid crystalline supramolecular polymers. They discovered that pH and salt concentration govern intermolecular interactions among self-assembled structures, with lower charge densities on supramolecular polymers and higher charge screening from the electrolyte, resulting in higher viscosity inks using a combination of experimental techniques and molecular dynamics simulations [193]. Printing of the supramolecular networks may lead to phase separation due to shear-induced in the process. Rupp et al., in their research, found that the printability of the two poly(isobutylene) [M_n_ = 8500–16,000 Da] can be enhanced by utilizing the nanocomposite form with silica nanoparticles with no phase separation [194].

The 3D-printed biomaterials for tissue regeneration need to undergo conformational changes according to the tissue structure with time. So, there has been a trend of 4D printing, utilizing the 3D-printed materials which could respond to the external stimuli or the internal tissue environment and transform over time. Many supramolecular systems could be used to print the cell-laden constructs and can be loaded with drugs or biomolecules and can be sensitized using various stimuli such as temperature, pH, electric fields, magnetic fields, and light to release them into the tissue environment [195].

## 8. Conclusions and Future Perspectives

Supramolecular systems which rely on non-covalent interactions are preferred for their unique properties such as thermodynamically reversible nature, self-healing properties, functionalization into bioactive materials, incorporation of stimuli responsivity, and others for tissue engineering. However, the challenge lies in the lack of robust materials that can be employed.

Bio-macromolecule-based supramolecular systems have resolved many issues that pertain to biocompatibility, but they are tough to produce at a larger scale with consistent properties. Synthetically derived supramolecular systems, on the other hand, have reduced batch-to-batch variations, and offer additional tailoring opportunities such as chemical signaling, delivery modulation, and others. The reproducibility and robustness of the preparation techniques affects the carrier capacity of these delivery systems and the interference with many material, formulation and process variables throughout the manufacturing and storage remains a bottleneck for these systems.

The preparation techniques are usually complicated and involve complex procedures that could be handled only by scientists who are well versed in polymer chemistry and medicine fields. The techniques are also required to be improved to increase the yield output and the rate of the reactions. Novel techniques need to be developed which can make scaffolds from a combination of the macromolecules which provide an easy translation into the healthcare industry. The use of technologies such as 3D printing, molecular simulations, and combinations thereof can further help the process of scalability and applicability in this regenerative medicine. The improvement in the accessibility of these systems will revolutionize the regenerative medicine field.

Even though there are many systems were successful in vitro and in vivo models, the clinical translation is still challenging. The clinical studies require the safety and efficacy to be evaluated and to take the long-term toxicity studies into consideration. Most of the polymers that have been synthesized are new and would require quite a large amount of significant clinical safety data in healthy volunteers before testing their efficacy on the patients, which also remains as a big hurdle for commercializing these novel therapeutic technologies.

Moving forward, these supramolecular systems may not only be limited to tissue regeneration but also would be applied to other ailments and for personalized medicine with the combination of medical devices and nanotheranostics. With the research on supramolecular systems increasing exponentially, many more advancements are anticipated in this emerging domain.

## Figures and Tables

**Table 1:** Characterization techniques and information obtained.

Characterization Technique	Evaluated Parameters	Information Obtained
**Rheology**	Elastic storage modulus (G’), elastic loss modulus (G”),	Stability of the supramolecular gel at induced strain and temporal variation. Structural assembly of the monomers [109,110]
**Chromatography**	Retention or exclusion time	The average molecular weight of the monomers [108]
**Spectroscopy:**		
Infrared	Vibrations of atoms and shifts in the characteristic peaks	Non-covalent interactions that appear during sol–gel transition/supramolecular assembly [111]
UV/Visible	Absorption wavelengths	Quantitative determination of non-covalent interactions that appear during sol–gel transition/supramolecular assembly [53,112,113]
Fluorescence	Loss of vibrational energy in the form of fluorescence	Quantitative determination of non-covalent interactions [114,115,116]
Nuclear magnetic resonance	Chemical shift, coupling constant, line widths, peak integral, relaxation time, and the nuclear Over Hauser effect (NOE)	Chemical structure and formation of non-covalent interactions in cavitands [56,117,118,119,120,121,122,123]
Mass spectrometry	Mass-to-charge ratio (*m*/*z*) of one or more molecules	Chemical structure and end groups identification [111,124,125,126]
Circular dichroism	The difference in absorbance of right- and left-circularly polarized light	The intra- and intermolecular interactions in chiral supramolecules with changes in temperature and time [115,126]
**Dynamic light scattering**	Intensity fluctuations during Brownian motion	Particle size [127,128,129,130,131]
**X-ray light scattering**	Intensities of X-rays scattered	Shape, conformation, and assembly of supramolecular complexes [77,125,132]
**Thermal analysis:** Isothermal titration calorimetry (ITC)	Various thermodynamic parameters—enthalpy (ΔH), entropy (ΔS), free energy (ΔG)	Binding affinity between the molecules and stoichiometry
Differential scanning calorimetry	Heat flow	Sol–gel transition enthalpy [130,133]
**Microscopy**	Topography of materials	Shape and size of the supramolecular delivery systems [77,111,118,134,135,136,137]

**Table 2:** Research on stability and biocompatibility.

Type of Tissue	Type of Supramolecular System	Application
Nervous tissue	Peptide-based hydrogels	Wang et al. proposed self-assembling peptides RADA16 with osteopontin-derived angiogenic motif SVVYGLR that helped in the angiogenesis and promoted the reconstruction of the neural tissue and recovery of the reflexive responses to the motion in a zebrafish model [138]
		Ma et al. proposed a self-assembling peptide hydrogel named Slanc with chemically synthesized oligopeptide sequence K(SL)3RG(SL)3K–G–KLTWQELYQLKYKGI with vascular endothelial growth factor. These hydrogels were first checked for the cytocompatibility using in vitro methods and then checked in fluid percussion injury models in vivo in Sprague–Dawley rats. The in vivo results have shown angiogenesis, neuroprotection and axonal outgrowth around the hydrogels indicating regeneration of the brain injury [139].
	Nucleic acid-based hydrogels	Yuan et al. made a highly permeable supramolecular DNA hydrogel matrix with homologous neural stem cells to regenerate severe spinal cord injury. The hydrogels were tested using in vivo rat models and have been evaluated for the migration, proliferation, and differentiation of the stem cells. It was found that the hydrogel-treated groups showed regeneration in the form of newborn oligodendrocytes and the myelin structure regeneration [140].
	Cyclic oligomer-based hydrogels	An injectable composite hydrogel made from gelatin acrylated β-cyclodextrin polyethylene glycol was prepared to carry motor neurons from embryonic stem cells by Wang et al. In their research, they demonstrated neuroinflammation response to the transplanted composite gels and a functional recovery in the behavior of the spinal cord injured rat models [141].
Cardiovascular tissue	Supramolecular peptide nanofibers	A zymogen activator peptide Nap-FFEG-IVGGYPWWMDV which can activate the hepatocyte growth factor precursor (pro-HGF) is made into self-assembled nanofibers to perform anti-apoptosis and pro-angiogenesis. This has helped to demonstrate regeneration of the infarct area of the heart affected due to acute myocardial infarction in the adult male C57 BL/6 mice [142]
		In another similar example, a self-assembling peptide Nap-pD-E7, that enables a sheet of mesenchymal stem cells, was isolated from the bone marrow to form cell spheroids. The peptide loaded with MSC spheroids is then checked in vivo in myocardial infarction-induced mice models. It was seen that the phosphopeptide promoted the paracrine effect and lead to neovascularization [143].
	Nucleic acid subunit-based systems	Growth factors are incorporated into supramolecular ureidopyrimidinone (Upy) hydrogel exhibiting sol–gel behavior that forms a gel at neutral pH. Intramyocardial delivery in a porcine model of myocardial infarction showed improved blood flow and the formation of new cardiomyocytes [144]. A similar study using chain extended ureidopyrimidinone polymers was functionalized with heparin and Interleukin 4 for in-situ arterial tissue regeneration in rat models [145].
Renal system	Supramolecular self-assembly β-sheet peptide hydrogel	A functional peptide-based scaffold containing Naphthalene (Nap) covalently conjugated to a short D-form peptide (Nap-DFDFG) and C domain of insulin-like growth factor-1 (IGF-1C) has been prepared to deliver the human placenta-derived mesenchymal stem cells. These hydrogels showed endogenous regeneration and improved blood flow when tested in a murine acute kidney model [146]
	Peptide nanofibers	Nanofibers derived from Arg-Gly-Asp peptides adhere readily to the integrins derived from the extracellular vesicles from the mesenchymal stem cells (MSCs). This system, when intrarenally injected into the acute kidney-injured rat models, was observed to determine an increase in proliferation, autophagy, and renal function [147]. The same research group also tested a hydrogel made by the combination of Tyr-Ile-Gly-Ser-Arg and Arg-Gly-Asp peptides and combined it with a biocompatible biotin-D-amino acid for carrying MSCs. The group reported the enhanced paracrine function of the MSCs in the acute kidney injured rat models [148].
	Cyclic oligomers	Cheng et al. proposed hypoxia-sensitive Azocalix(5)arenes co-assembled with mesenchymal stem cell-derived extracellular vesicles for targeted therapy in kidney injury. They reported the inhibition of HIF-1α expression in hypoxic renal tubular epithelial cells (TECs). This delivery system could be used further for delivering therapeutic and diagnostic agents [149].
Digestive system	Cyclic oligomers	In the cases of treatment of type 1 diabetes, the transplanted pancreatic islets suffer from oxidative stress and inflammation. Delivery of bilirubin with the help of poly lysine conjugated cyclodextrin was attempted in a diabetic mice model. This system helped in the reduction of the oxidative stress and inflammation, promoted angiogenesis, and strengthened the function of the transplanted islets [150].
	Amphiphilic block copolymers	For tissue repair in gastric environments, Wang et al. developed an anti-biofouling and biocompatible hydrogel based on ABA triblock copolymers. This hydrogel was checked in the in vivo gastric perforation repair model, and the results suggested a constrained inflammation and an increase in the vascular density. The reduced inflammation was explained due to the anti-biofouling capacity, which is the ability to prevent the accumulation of the microbes during the wound healing process, which makes it an ideal material for postoperative wound dressing for tissue regeneration [151].
Muscle/Connective	Peptide nanofibers	In the case of muscle regeneration, the basal lamina plays a significant role. The laminin mimetic peptide nanofibers lauryl-VVAGKKIKVAV-Am mimics the muscle basal lamina environment. It significantly promoted satellite cell activation in skeletal muscle and myogenic differentiation and accelerated myofibrillar regeneration in the in vivo acute muscle injury model in Sprague–Dawley rats [152].
		In conditions such as sarcopenia, which is described by a rapid loss of muscle mass and function, delivery of growth factor-like Insulin-like growth factor -1 (IGF-1) has proven beneficial. Shang et al. attempted muscle repair by using IGF-1mimicking peptide sequence Nap-FFGSSSR which forms a supramolecular hydrogel, which could promote myoblast proliferation and promoted muscle regeneration in glucocorticoid-induced sarcopenia rat models. Further RNA sequencing was performed to elucidate the similarity in the activation of the Akt signaling pathway by IGF-1 to that of the peptide hydrogel [153].
	Nucleic acid subunit-based systems	Mori et al. described a supramolecular material with polycarbonate and ureidopyrimidinone functionalized with UPy-modified cyclic arginine-glycine-aspartic acid (cRGD) peptide additives. This material promoted myogenesis and neovascularization in the rat hernia model accelerating the tissue growth and regeneration of the abdominal wall [154].
Bone/Cartilaginous	Peptide amphiphiles	Histidine-containing peptides incorporated with a dicarboxylic acid-like succinic acid have been used for three-dimensional cell culture using a mouse fibroblast cell line. In vitro studies revealed the growth and nourishment of cells in the presence of the peptide gel [155,156]
	Cyclic oligomer-based systems	β-cyclodextrin—gellan gum complex hydrogel was developed to enhance the affinity of the anti-inflammatory drug dexamethasone known for improving the chondrogenesis and decreasing the inflammatory response in the cartilage defected rabbit model [59]. Another such example was described in the research by Jeong et al., where mesenchymal stem cells were encapsulated in β-cyclodextrin modified hyaluronic acid and adamantane modified Hyaluronic acid.
	Nucleic acid-based systems	Yan et al. proposed a DNA-based hydrogel with isolated mesenchymal stem cells (MSC) and tested it in vitro to check for cell proliferation and chondrogenic differentiation. Upon confirmation of the differentiation, the hydrogels were injected into severe osteoarthritis rabbit models which supported the MSC in a high-friction environment and showed signs of cartilage regeneration [156].
	Lipid-based systems	Molecules such as Rhein and other therapeutic agents which possess chondroprotective action are very poorly bioavailable when taken through the oral route. Ebada et al., in their research, made cationic solid lipid nanoparticles entrapped with rhein and which could be injected directly into the articular joints. The lipid-based systems proved efficient in inhibition of inflammation, and cartilage deterioration in the Monoiodoacetate induced arthritic rat models [157].
Dental	Peptide-based hydrogels	Siddiqui et al. made a self-assembling peptide SLan angiogenic target peptide with K(SL)6K–G–KLTWQELYQLKYKGI sequence for dental pulp revascularization. They checked for biocompatibility with subcutaneous administration of the peptides to the female wistar rats and then in adult beagle dogs as pulp revascularization models. The growth factor mimetic peptide was found to be the material of choice for tissue regeneration, promoting angiogenesis in cases of pulpectomy [158].
		Biocompatible hydrogelator Nap-Phe-Phe-Tyr-OH (NapFFY) was co-assembled with stromal cell-derived factor-1 (SDF-1) and bone morphogenetic proteins (BMPs) and studied for periodontal bone regeneration by Tan et al. The team observed that the hydrogel has increased the cell proliferation of the endogenous bone marrow mesenchymal stem cells due to the SDF-1, and in vivo models of the critical-sized periodontal bone defect models of maxillae in rats suggested the accelerated osteogenesis [159].
	Nucleic acid subunit-based systems	Wang et al. used a monomeric nucleoside molecular gelator 2-amino-2′-fluoro-2′-deoxyadenosine, which is self-healable, shear-thinning, and injectable to the tooth sockets directly. After checking for the biocompatibility, the team checked the anti-bacterial activity against the streptococcus mutans and the porphyromonas gingivalis and observed reduced inflammation at the sites of application in the in vivo rat models, thereby helping in the regeneration of the tooth socket [160].
Skin	Nucleic acid subunit-based systems	For accelerated wound healing and skin regeneration, guanosine quartet hydrogels loaded with recombinant human-sourced collagen (G4-RHC) are used as medical patches. The RHC that is integrated into the gel engages macrophages and fibroblasts at the injury site and supports the formation of new connective tissue for skin regeneration [161].
	Peptide amphiphile nanofibers	To heal wounds made by burns, bioactive Arg-Gly-Asp-Ser (RGDS) modified peptide amphiphilic gels seeded with thermally damaged fibroblasts and human umbilical vein endothelial cells were used in a rat burn model. Pathological and histological examinations have been done in the injured area, which showed significant re-epithelialization and capillary formation supporting the burn wound closure [162].
	Peptide hydrogels	Jian et al. designed a platelet-derived growth factor (PDGF) mimicking peptide by connecting a self-assembling motif derived from β-amyloid peptide and the PDGF epitope VRKIEIVRKK. Upon checking for cell proliferation of fibroblasts and keratinocytes and cell migration, the formulation was tested in a full-thickness skin wound model. The collagen disposition and the angiogenesis in the in vivo model support the finding of this material as a suitable biomaterial for chronic wound healing [163].
	Cyclic oligomer-based hydrogels	To enhance wound healing, epidermal growth factor (EGF) was delivered using supramolecular polysaccharide hydrogels consisting of cyclic oligomer β-cyclodextrin and azobenzene groups conjugated to hyaluronic acid chains. The formulation was tested on a full-thickness skin defect model and showed an increase in growth factor levels, granulation tissue formation, and angiogenesis [53].
		For wound healing, supramolecular hydrogels were made with cyclodextrin-modified gelatin and adamantane-modified hyaluronate, with encapsulated fibroblasts and conjugated with human growth factor. After checking for cytocompatibility and cell proliferation, the mice were injected with fibroblasts mixed with these hydrogels and checked for skin regeneration for 21 days. The research group found that fibroblast proliferation from the hydrogels has helped in angiogenesis and skin regeneration [164].

## Data Availability

Not applicable.

## Abbreviations

ECM	Extracellular matrix
HDL	High density lipoproteins
GML	Glyceryl monolaurate
DDG	1-O-dodecyl- rac -glycerol
BMP-2	Bone morphogenetic protein 2
Apo A1	Apolipoprotein A1
LXR	Liver X receptor
Fmoc	Fluorenyl methoxycarbonyl
A	Adenine
G	Guanine
U	Uracil
T	Thymine
C	Cytosine
PEG	Polyethylene glycol
ChS-F	Furfuryl amine grafted chondroitin sulfate
PAMAM	Polyamidoamine
HA	Hyaluronic acid
RGD	Arginyl glycyl aspartic acid
MSC	Mesenchymal stem cells
CD	Cyclodextrins
TGF-β1	Transforming growth factor beta 1
CB[n]	Cucurbit(n)urils
DNA	Deoxyribonucleic acid
RNA	Ribonucleic Acid
PLLA	poly-L-lactic acid
PCL	polycaprolactone
PLA	polylactic acid
PEO	polyethylene oxide
BCP	Block co-polymers
CMC	Critical micellar concentration
UV	Ultraviolet
NOE	Nuclear Over Hauser effect
DOSY	Diffusion-ordered NMR spectroscopy
NOESY	Nuclear Over Hauser effect spectroscopy
ROESY	Rotating frame nuclear Over Hauser effect spectroscopy
DLS	Dynamic light scattering
DSC	Differential scanning calorimetry
SEM	Scanning electron microscopy
TEM	Transmission electron microscopy
AFM	Atomic force microscope
HGF	hepatocyte growth factor
IGF-1	Insulin-like growth factor -1
TEC	tubular epithelial cells
PDGF	platelet-derived growth factor
EGF	epidermal growth factor
DOPA	3,4-dihydroxyphenylalanine
ROS	Reactive oxygen species
MD	molecular dynamics
AMBER	Assisted Model Building with Energy Refinement
CHARMM	Chemistry at Harvard Macromolecular Mechanics
OPLS	optimized potentials for liquid simulations
GROMOS	Groningen Molecular Simulations
